# Effect of Poloxamer 407 as a carrier vehicle on rotator cuff healing in a rat model

**DOI:** 10.1186/1749-799X-9-12

**Published:** 2014-03-01

**Authors:** Soung-Yon Kim, Soo-Won Chae, Juneyoung Lee

**Affiliations:** 1 Department of Orthopaedic Surgery, Hallym University Kangnam Sacred Heart Hospital, 948-1, Daerim-1 Dong, Yeongdeungpo-gu, Seoul 150-950, South Korea; 2 Department of Mechanical Engineering, Korea University, Anam-Dong, Seongbuk-Gu, Seoul 136-713, South Korea; 3 Department of Biostatistics, Korea University College of Medicine, Anam-Dong, Seongbuk-Gu, Seoul 136-705, South Korea

**Keywords:** Rotator cuff, Carrier vehicle, Healing, Histological analysis, Biomechanical testing

## Abstract

**Background:**

*In vivo* studies showing the effects of biologic healing-promoting factors on tendon-to-bone healing after rotator cuff repair have focused only on biologic healing-promoting factors and have not taken into consideration the effect of the carrier vehicle. Moreover, most studies have evaluated the healing process using different carrier vehicles, each of which may have specific effects on tendon healing. This may explain the large variability seen in outcomes in research studies. In this study, we investigated the effects of Poloxamer 407 as a carrier vehicle on rotator cuff healing at the repair site and compared it with those of a collagen sponge, which is a commonly used carrier vehicle.

**Methods:**

Fifty-seven adult male Sprague–Dawley rats underwent detachment and immediate repair of the bilateral supraspinatus tendons. Rats were randomly assigned to three groups: repair only, repair with collagen sponge, and repair with Poloxamer 407. The repairs were evaluated at 1, 2, 4, and 8 weeks after surgery with histological analysis and biomechanical testing.

**Results:**

At 4 weeks, more cellular organization, a greater number of collagen fibers, and increased maturity of collagen fibers were observed in the repair with Poloxamer 407 group than in the other groups. The repair with collagen sponge group had delayed development and collagen fiber maturation. Significant differences in the biomechanical properties were found between groups at 4 weeks. Stiffness in the case of the repair with Poloxamer 407 group was significantly higher than that in the repair with collagen sponge group. The modulus was significantly lower in the repair with collagen sponge group than in the repair only group. However, the use of Poloxamer 407 versus the collagen sponge did not significantly affect the biomechanical properties of the repaired tendons at 8 weeks.

**Conclusions:**

Carrier vehicles may have differing effects at the early stages of rotator cuff healing. The use of Poloxamer 407 as a carrier vehicle may be useful for promoting the early stages of healing and for maintaining the initial biomechanical properties of the repaired rotator cuff tendon.

## Background

Most rotator cuff tears are treated surgically; however, recent research has shown high re-rupture rates of 13%–94% [1-3]. Rotator cuff repair often requires tendon-to-bone healing, and previous studies have noted that failure of rotator cuff repair is a challenging clinical problem [3-5]. Several studies have focused on methods to enhance the healing process after rotator cuff repair, and biological approaches have improved tendon healing in animal models [6-10]. The healing capacity of various factors, including growth factors (i.e., vascular endothelial growth factor and platelet-derived growth factor), mesenchymal stem cells, platelet-rich plasma, and bone morphogenetic proteins (BMPs), has been tested in animals [11,12].

In animal studies, these biologic healing-promoting factors have been delivered through various carrier vehicles such as type I collagen sponge, fibrin glue, and hyaluronan sponge to increase their retention at the repair site, localize tissue repair, and, in some instances, provide a scaffold for cellular ingrowth [10,13]. However, most studies have focused only on the biologic healing-promoting factors and not taken into consideration the effect of the carrier vehicle. In addition, studies have evaluated the healing process using different carrier vehicles, each of which may have specific effects on tendon healing and lead to a large variability in outcomes.

Poloxamer 407 is a triblock polymer that exhibits concentration-dependent reverse thermal gelation, in which aqueous solutions are liquid at low temperatures (-10°C) and form semisolid gels at body temperature. This characteristic is potentially useful for sustained release of injectable drugs. Local injectable Poloxamer 407 formulations have been shown to promote slow and sustained drug release directly at the site of interest [14,15]. Studies have also demonstrated that Poloxamer 407 can carry a sufficient amount of drug and shows good tolerability, biodegradability, non-toxicity, water solubility, and controlled release [16]. Animal studies also support the applications of Poloxamer 407 as a reversible thermosensitive microvascular clamp for surgery [17] and as an antiadhesive material for laminectomy procedures [18].

We aimed to investigate the effects of a carrier vehicle on rotator cuff healing at the repair site by using Poloxamer 407 (polymer) and a collagen sponge, both of which are commonly used as carrier vehicles. We evaluated rotator cuff healing rates at the tendon-to-bone repair site after surgical repair with either collagen sponge or Poloxamer 407 and examined whether tendon-to-bone remodeling after surgical repair of the supraspinatus tendon was affected by local application of a collagen sponge or Poloxamer 407. We hypothesized that each would have different effects on tendon-to-bone healing, biomechanical strength, and remodeling of the tendon repair. We also hypothesized that Poloxamer 407 (polymer) would have a better effect on early tendon-to-bone healing process than collagen sponge.

## Methods

### Animals

Bilateral shoulders of 57 adult male Sprague–Dawley rats (mean body weight, 457 g; range, 410–500 g) were randomly allocated to three groups: 19 rats (38 shoulders) underwent tendon-to-bone repair only, 19 (38 shoulders) underwent tendon-to-bone repair with application of the collagen sponge carrier vehicle to the repair site (repair + CS), and 19 (38 shoulders) underwent tendon-to-bone repair with application of the Poloxamer 407 carrier vehicle to the repair site (repair + P407). Principles of laboratory animal care (NIH publication no. 86–23, revised 1985) were followed throughout the study, and approval for the study was obtained by the institutional animal care and use committee (#KIACUC-09-0016).

### Surgical procedure

**Figure 1:**
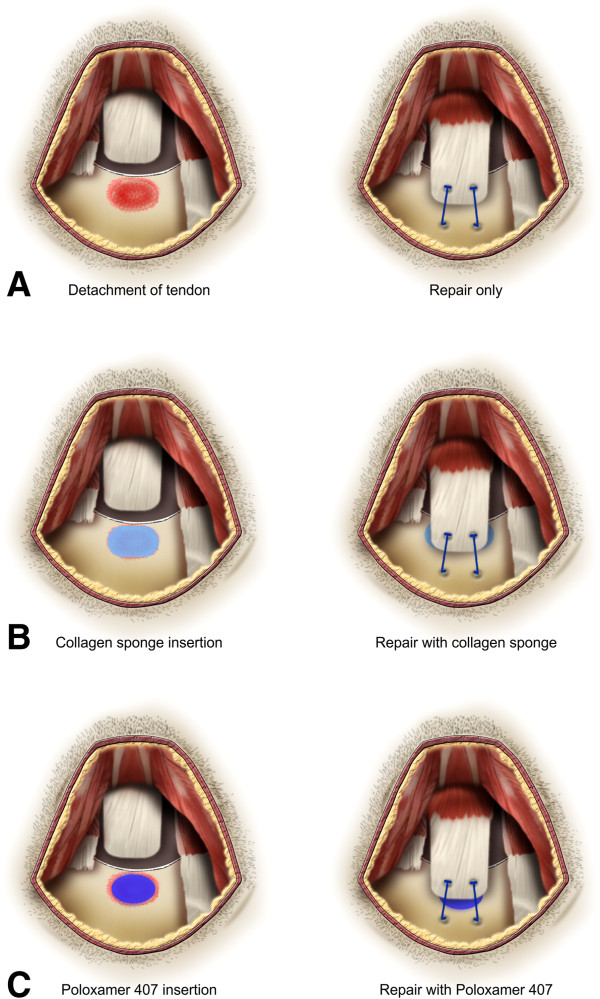
**Illustration of rotator cuff repair in a rat model. (A)** Detachment of the supraspinatus tendon followed by repair without any carrier vehicle (repair only). **(B)** Collagen sponge was applied at the interface of the detached tendon and footprint in the bone before the tendon was repaired (repair + CS). **(C)** Poloxamer 407 gel was injected between the detached tendon and footprint in the bone before the tendon was repaired (repair + P407).

Bilateral shoulder surgery was performed under sterile conditions. General anesthesia was administered with inhaled enflurane gas (Gerolan, Choongwae Pharmaceutical, Seoul, South Korea; in an oxygen carrier by using a nose cone) and 80 μL/g intraperitoneal Zoletil (Virbac, Carros, France). Using a technique similar to the established surgical model of supraspinatus tendon detachment in rats [19], a 2-cm vertical skin incision was made over the lateral aspect of the acromion. The deltoid muscle was dissected sharply from the acromion and divided distally. The supraspinatus tendon was exposed and then divided from the other rotator cuff tendons before sharp detachment at its insertion on the greater tuberosity using a scalpel blade. The distal fibrocartilage remaining on the greater tuberosity was removed, and decortication was performed using a dental burr. A 0.5-mm drill hole was created transversely in an anteroposterior direction through the proximal part of the humerus. The tendon was grasped using a double-armed 5–0 Prolene suture (Ethicon, Somerville, NJ, USA) using a technique similar to the Mason-Allen method. The suture was passed through the drill hole, and the tendon was re-apposed to its anatomic site (Figure 1A). In the repair + CS group, before tightening the suture, a type I collagen sponge (Bioland, Chungnam, South Korea) was shaped to cover the detached tendon area of the supraspinatus footprint consistently (3 × 3 × 1 mm) and applied at the interface of the tendon and bone. Sutures were tied over the collagen sponge, affixing it to the interface in a compressive manner (Figure 1B). In the repair + P407 group, 10 μL of the gel formed Poloxamer 407 (BASF, Nienburg/Weser, Germany) with a particle size of 10*λ* and was injected between the tendon and the supraspinatus footprint in the bone before tightening the suture (Figure 1C). The suture was then tightened, securing the tendon down to the bone. The deltoid and trapezius muscles were re-approximated, and the skin was closed. After surgery, each rat was allowed unrestricted postoperative cage activity without immobilization.

### Sample collection

**Figure 2:**
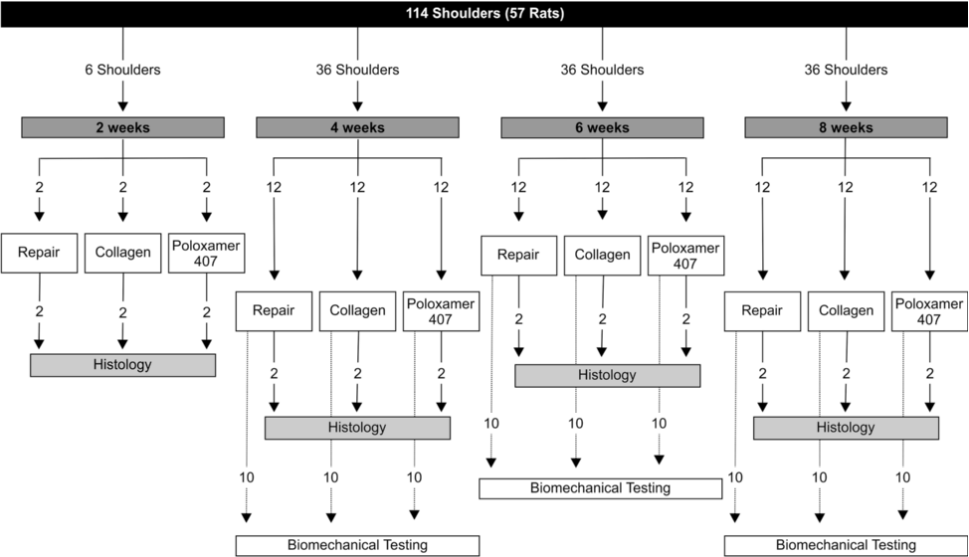
**Flow diagram representing the study design.** The shoulders of the rats were divided into three groups and evaluated at four time points.

The rats were euthanized by intracardiac injection of sodium phenobarbital at 1, 2, 4, or 8 weeks after surgery. Two specimens per group were prepared for histological analysis at 1, 2, 4, and 8 weeks after surgery, and 10 specimens per group were prepared for biomechanical testing at 2, 4, and 8 weeks after surgery. Each shoulder was dissected to isolate the supraspinatus tendon-humerus specimen, and the tendinous portion of the supraspinatus was isolated by removing all soft tissues (Figure 2).

### Histological analysis

Specimens were fixed overnight in 4% paraformaldehyde and decalcified in 14% ethylenediamine tetraacetic acid. Specimens were embedded in paraffin, sectioned at 5 μm, and dried for 1 h at 60°C, after which they were stained with hematoxylin and eosin (cell morphology, inflammation, cellularity, vascular proliferation, and fibroblast proliferation), toluidine blue (fibrocartilage formation and tidemark), and Picrosirius red (collagen organization). Picrosirius red staining and examination with monochromatic polarized microscopy was used for analyzing collagen organization at the tendon-to-bone repair site [20]. We evaluated these histologic parameters with the grading criteria used for the tendon-to-bone maturation scoring system described by Ide et al. [21]. Immunohistochemical analysis of angiogenesis and capillary formation was performed on 5-μm sections cut from formalin-fixed paraffin-embedded tissue that was stained with rabbit monoclonal anti-CD34 (EP373Y; 1:50, Abcam, Cambridge, MA, USA). The visualization system used was the BenchMark XT (Ventana, Tucson, AZ, USA) with heat-induced epitope retrieval (CC1 solution, Ventana, Tucson, AZ, USA). Sections were incubated with primary antibodies for 32 min at 37°C. Staining was detected with the ultraView Universal DAB detection kit (Ventana). Microvessels were counted in 10 separate × 400 magnification fields. The three fields with the highest angiogenesis out of the 10 areas were taken as the microvessel density (MVD). The lower third MVD was considered mild, the middle third as moderate, and the highest as severe according to the relative comparison of all specimens. Three to five sections per group were evaluated based on section quality. The sections were evaluated by an independent experienced blinded pathologist.

### Biomechanical testing

**Figure 3:**
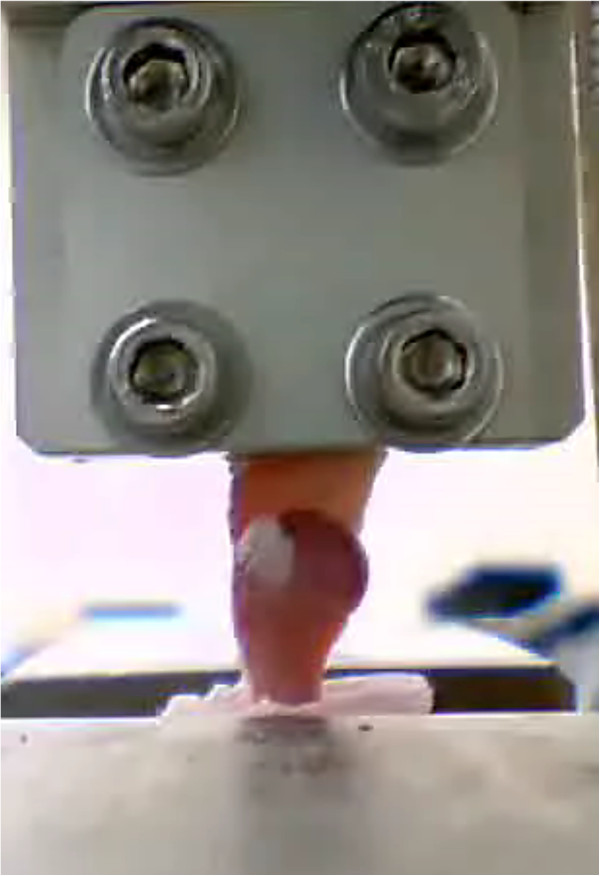
**Biomechanical testing.** Supraspinatus tendon-humerus specimen was mounted on a material testing system by using a specially designed jig.

Supraspinatus tendon-humerus specimens were kept hydrated in saline solution during preparation and were wrapped in gauze, wetted with saline solution, covered with plastic film, and stored at -60°C until the start of the biomechanical testing. Tendon-to-bone repair site thickness and width were measured using digital calipers after thawing at room temperature. The cross-sectional area of the repair site was calculated by thickness and width, assuming an elliptical cross Section. A specially designed jig was prepared for gripping and mounting the proximal part of the supraspinatus tendon portion of the specimen for biomechanical testing. The remaining humeral portion of the specimen was embedded in a plastic tube with resin (Lang Dental Manufacturing Co., Wheeling, IL, USA) for mounting on a material testing system (model 5882; Instron, Norwood, MA, USA). Supraspinatus tendon-humerus specimens were tested under uniaxial tension at room temperature in line with the pull of the supraspinatus tendon using a material testing system (Figure 3). Testing was done at room temperature, and specimens were kept hydrated by spraying with saline solution. The biomechanical testing protocol was similar to that described by Galatz et al. [22]. After preloading the specimen to 0.2 N, tendons were preconditioned for 5 cycles to 0.38 mm of displacement at a rate of 0.1 mm/s and held for 300 s. They were then tested to failure at a rate of 0.1 mm/s. Maximum load versus extension was recorded for each specimen. Extension measurements were determined by machine displacement. Elastic properties, such as stiffness and modulus, were calculated using linear regression from the near-linear region of the load–displacement and stress–strain curves, respectively. Maximum stress was calculated by dividing the maximum load by the cross-sectional area. Stiffness was determined by determining the slope of the load versus the extension curve in the linear region following toe-in.

### Statistical methods

Biomechanical results were compared using a one-way ANOVA for groups (repair only, repair + CS, and repair + P407) and time (2, 4, and 8 weeks) followed by Tukey's multiple comparison test. Primary endpoints included maximum load, maximum stress, modulus, and stiffness in each group at each time point. Data are presented as means ± standard deviations. *p* < 0.05 was considered statistically significant. Histology-based results were qualitative in nature and were not statistically compared.

## Results

### Histology

**Figure 4:**
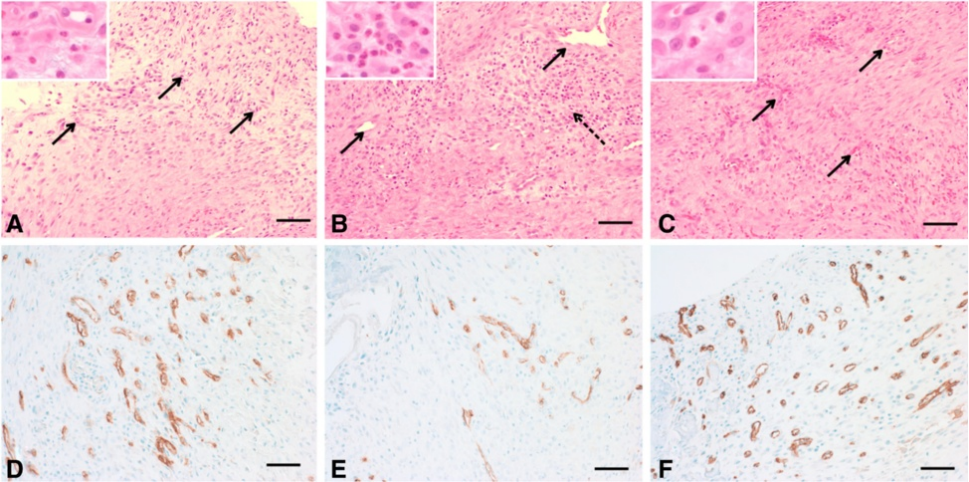
**Histological findings at 1 week after tendon-to-bone repair. (A)** Repair only. **(B)** Repair + CS. **(C)** Repair + P407 (hematoxylin and eosin; magnification, ×100, *insets* × 400). The inflammatory reaction was more marked in the repair + CS specimens. *Arrows* indicate angiogenesis, and *dashed arrow* indicates inflammatory cells. *Scale bars*, 100 μm. **(D)** Repair only. **(E)** Repair + CS. **(F)** Repair + P407 (immunohistochemical staining with rabbit monoclonal anti-CD34, ×200). Vascular endothelium was stained with a *brown color*. Capillary proliferation was observed less in the repair + CS specimens than in the repair only and repair + P407 specimens. *Scale bars*, 200 μm.

**Figure 5:**
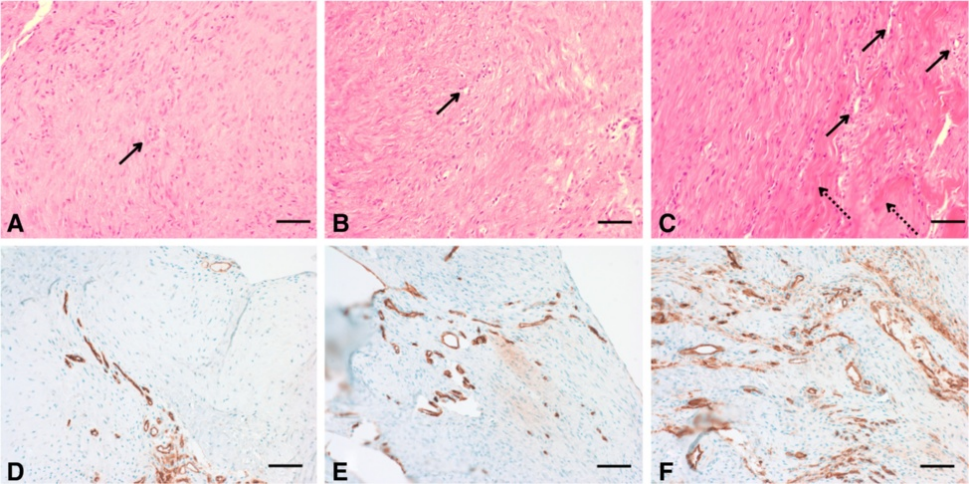
**Histological findings at 2 weeks after tendon-to-bone repair. (A)** Repair only. **(B)** Repair + CS. **(C)** Repair + P407 (hematoxylin and eosin; magnification, ×100). Capillary proliferation as well as development and maturity of connective tissue were greater in the repair + P407 specimens than in the repair only and repair + CS specimens. *Arrows* indicate angiogenesis, and *dashed arrows* indicate mature collagen fibers. *Scale bars*, 100 μm. **(D)** Repair only. **(E)** Repair + CS. **(F)** Repair + P407 (immunohistochemical staining with rabbit monoclonal anti-CD34, ×200). Capillary proliferation was stained with a *brown color* and was marked in the repair + P407 specimens than in the repair only and repair + CS specimens. *Scale bars*, 200 μm.

**Figure 6:**
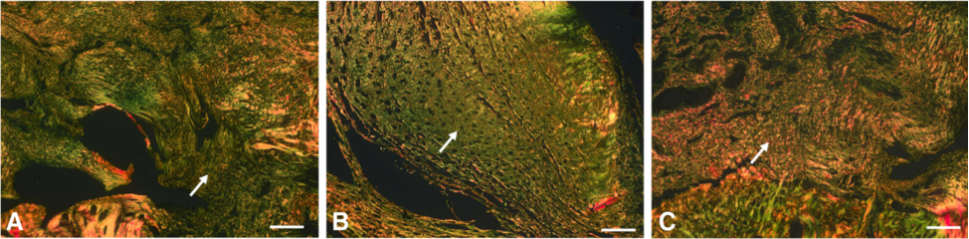
**Histological findings 4 weeks after tendon-to-bone repair. (A)** Repair only. **(B)** Repair + CS. **(C)** Repair + P407 (Picrosirius red; magnification, ×100). Immature collagen fragments are stained in *green*. As the maturation and organization of collagen fibers increased, the green staining changed to *yellow* and subsequently to *orange* at the repaired tendon-to-bone site. More cellular organization, more collagen fibers, and increased maturity of collagen fibers were observed in the repair + P407 specimens than in the repair only and the repair + CS specimens. *White arrows* indicate collagen fibers. *Scale bars*, 100 μm.

**Figure 7:**
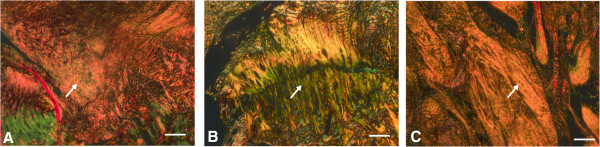
**Histological findings 8 weeks after tendon-to-bone repair. (A)** Repair only. **(B)** Repair + CS. **(C)** Repair + P407 (Picrosirius red; magnification, ×100). Immature collagen fragments are stained in *green*. As the maturation and organization of collagen fibers increased, the green staining changed to *yellow* and then *orange* at the repaired tendon-to-bone site. The amount and maturity of collagen fibers were similar in the repair only and the repair + P407 specimens. Fewer and less mature collagen fibers were observed in the repair + CS specimens. *White arrows* indicate collagen fibers. *Scale bars*, 100 μm.

Gross inspection of all the specimens during dissection revealed that the tendon-to-bone repair site was enlarged and thickened. The inserted carrier vehicles, collagen sponge or Poloxamer 407, were not visible and did not definitively discriminate from other tissues at the time of dissection. At 1 week, the inflammatory reaction was more marked in the repair + CS specimens than in the repair only and repair + P407 specimens and showed high cellularity and capillary proliferation (Figure 4). At 2 weeks, fewer inflammatory cells were seen in all specimens than at 1 week, and a tidemark began to be evident. Capillary proliferation and development as well as connective tissue maturity were marked in the repair + P407 specimens than in the repair only and repair + CS specimens (Figure 5). At 4 weeks, there were distinct decreases in cellularity and vascularity in the repair + P407 specimens. There were more cellular organization, a greater number of collagen fibers, and increased maturity of collagen fibers in the repair + P407 specimens than in the other specimens (Figure 6). At 8 weeks, the tendon at the repair site showed well-organized collagen fibers oriented in line with the tensile pull of the tendon. Fibers were greater in number, maturity, and organization at 8 weeks than at 4 weeks. Similar findings for development and maturation of collagen fibers were observed in the repair only and repair + P407 specimens. However, fewer and less mature collagen fibers were observed in the repair + CS specimens (Figure 7).

### Biomechanics

**Figure 8:**
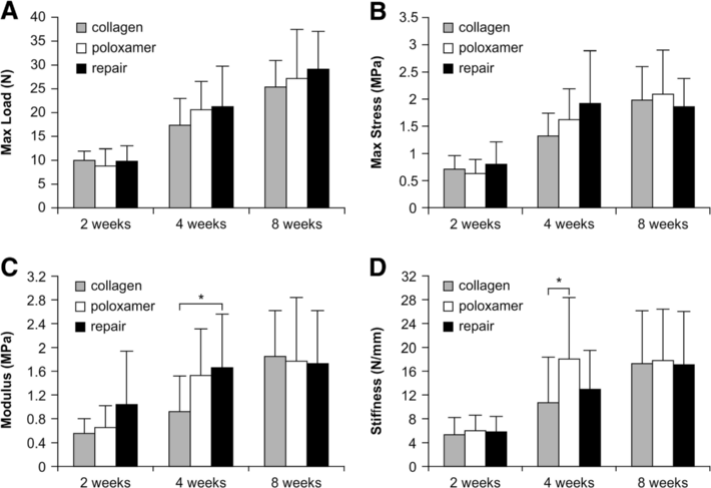
**Graphs representing biomechanical findings in the three groups. (A)** Maximum load (N) was not significantly different within each group at any time point. **(B)** Maximum stress (MPa) was not significantly different within each group at any time point. **(C)** The modulus (MPa) of the repair + CS specimens was significantly lower than that of the repair only specimens at 4 weeks (*p < 0.05). The modulus increased significantly in the repair only and repair + P407 specimens between 2 and 4 weeks. **(D)** Stiffness (N/mm) of the repair + P407 specimens was significantly higher than that of the repair + CS specimens at 4 weeks (*p < 0.05) and increased significantly from 2 to 4 weeks after surgery.

All specimens exhibited a comparable gradual increase in maximum load, maximum stress, modulus, and stiffness over 8 weeks. All groups showed similar biomechanical properties at 2 and 8 weeks. At the 4-week time point, some of the biomechanical properties such as modulus and stiffness varied significantly between the groups. Maximum load (N; Figure 8A) and maximum stress (MPa; Figure 8B) were not significantly different between groups at any time point. The modulus (MPa) was significantly lower in repair + CS specimens than in repair only specimens at 4 weeks (*p* = 0.027; Figure 8C). The modulus increased significantly between 2 and 4 weeks in the repair only and repair + P407 specimens (*p <* 0.01; Figure 8D). Stiffness (N/mm) of the repair + P407 specimens was significantly higher than that of the repair + CS specimens at 4 weeks (*p* = 0.044) and increased significantly from 2 to 4 weeks after surgery (*p* < 0.01; Figure 8D). However, no significant differences were found between groups at 8 weeks (Figure 8D).

## Discussion

Clinical studies on rotator cuff repair have demonstrated a high rate of incomplete healing and gap formation between tendon and bone [1,2,23,24]. Re-tears and failure with continuity after rotator cuff repair occur in the early postoperative period [25,26], and the tendon may re-rupture from the repair site because of its inability to control the loads placed on it during the early postoperative period [27]. Therefore, enhancing tissue regeneration and maintaining repair strength during the early period after rotator cuff repair may decrease the re-tear or failure rates.

Several studies have suggested that biologic healing-promoting factors improve healing after rotator cuff tendon-to-bone repair, such as cartilage-derived morphogenetic protein 2 [9], osteoinductive growth factors (BMPs 2–7), transforming growth factor-β 1–3, and fibroblast growth factor (FGF) [10] and FGF-2 [21]. Different carrier vehicles have been used in these studies, including collagen sponge, fibrin glue/sealant, and hyaluronan sponge/paste, to increase local retention and slow the release of healing-promoting factors at the repair site. However, the influence of the various carrier vehicles on the rotator cuff tendon healing has not been reported, and little is known about the effect of the carrier vehicles used for delivery of healing-promoting factors on rotator cuff healing.

We evaluated the effect of two carrier vehicles on rotator cuff healing at the tendon-to-bone repair site: Poloxamer 407, the use of which has been extended to *in vivo* studies in various surgical specialties [14,16,18], and collagen sponge, which is a commonly used carrier vehicle. To assess the direct and measurable effect of these two carrier vehicles on rotator cuff tendon-to-bone healing, we applied a collagen sponge or Poloxamer 407 to the interface of the detached supraspinatus tendon and footprint in the bone rather than applying these carrier vehicles over the rotator cuff tendon-to-bone repair site.

Our histological and biomechanical results show that the initial tendon-to-bone healing process was affected by local administration of the carrier vehicle and that the healing process differed depending on the carrier vehicle used.

Among the biomechanical properties, stiffness of the repair + P407 group was significantly higher than that of the repair + CS group at 4 weeks. However, modulus and stiffness in the repair + CS group were significantly lower than those in the repair only or repair + P407 groups. These biomechanical results were consistent with histological findings. Development and maturity of collagen fibers were higher in the repair + P407 group than in the repair only and repair + CS groups at 2 and 4 weeks. The amount and maturity of collagen fibers were more distinct at 4 weeks in the repair + P407 group, and this was reflected by improved biomechanical properties. In the repair + CS group, fewer and less mature collagen fibers were observed than in the other groups even at 8 weeks. These data suggest that Poloxamer 407 exhibited an early healing process with respect to collagen fiber maturation compared with the collagen sponge. Based on our findings, we postulate that Poloxamer 407 as a carrier vehicle may be more useful to maintain the repair strength, particularly during the early stages of the healing period, than a collagen sponge. These initial increased material properties and healing process may decrease the risk of early re-tear failure after rotator cuff repair.

Our results confirmed our hypothesis that different carrier vehicles have different effects on the biomechanical properties and healing process of repaired rotator cuff tendons. Based on our histological and biomechanical findings, we suggest that the use of a carrier vehicle may delay or promote the early stage of tendon-to-bone healing and also affect the repair strength. The early stage of tendon-to-bone healing may be a critical period when re-tear might occur after rotator cuff repair.

The present study has some limitations. First, the surgical repair was performed for an acute rotator cuff tear rather than for degenerated and retracted tendon tears, which would have been more clinically relevant. Second, the use of a quadruped animal associated with a weight-bearing forelimb to model the human shoulder may be a limitation [28]. Third, this study was limited to 8 weeks postoperatively; therefore, the durability of these results cannot be evaluated and should be addressed. Additionally, we evaluated only the carrier vehicles and did not evaluate their optimal amounts, interactions, or effects when used with biologic healing-promoting agents such as growth factors, cytokines, and stem cells.

## Conclusions

The carrier vehicles used for healing-promoting agent retention have different effects on rat rotator cuff tendon-to-bone healing depending on the carrier vehicle used, suggesting that the use of a carrier vehicle alters tendon biology and mechanical strength, particularly during the early stage of healing. The use of Poloxamer 407 as a carrier vehicle may be useful for promoting the early stages of the healing process and for maintaining the initial biomechanical properties of repaired rotator cuff tendons. In the early healing stage, the biomechanical properties of the repaired tendon-to-bone insertion in the regenerated tissue of an *in vivo* rotator cuff were better with Poloxamer 407 compared to the collagen sponge, suggesting the probable feasibility of regenerating rotator cuffs using current tissue engineering techniques with this polymer.

## Competing interests

The authors declare that they have no competing interests.

## Authors' contributions

SYK contributed towards the study design, data acquisition, surgical procedures, manuscript preparation, and final approval. SWC and JL contributed towards the data acquisition and data analysis and participated in writing the manuscript. All authors read and approved the final manuscript.
